# Genetic identification of *Pid3-1* and its regulatory role in promoting blast resistance in rice

**DOI:** 10.1093/g3journal/jkag027

**Published:** 2026-02-08

**Authors:** Junhua Liu, Niqing He, Zhaoping Cheng, Shaojun Lin, Shiyuan Fu, Mingmin Wang, Fenghuang Huang, Salah F Abou-Elwafa, Nora M Al Aboud, Chengzhi Huang, Dewei Yang

**Affiliations:** Chongqing Three Gorges Academy of Agricultural Sciences, Wanzhou, Chongqing 404155, China; Institute of Rice, Fujian Academy of Agricultural Sciences, Fuzhou 350018, China; Institute of Rice, Fujian Academy of Agricultural Sciences, Fuzhou 350018, China; Institute of Rice, Fujian Academy of Agricultural Sciences, Fuzhou 350018, China; Institute of Rice, Fujian Academy of Agricultural Sciences, Fuzhou 350018, China; College of Agriculture and Plant Immunity Center, Fujian Agriculture and Forestry University, Fuzhou 350002, China; Institute of Rice, Fujian Academy of Agricultural Sciences, Fuzhou 350018, China; College of Agriculture and Plant Immunity Center, Fujian Agriculture and Forestry University, Fuzhou 350002, China; Chongqing Three Gorges Academy of Agricultural Sciences, Wanzhou, Chongqing 404155, China; Agronomy Department, Faculty of Agriculture, Assiut University, Assiut 71526, Egypt; Department of Biology, Faculty of Science, Umm Al-Qura University, Makkah 21955, Saudi Arabia; Chongqing Three Gorges Academy of Agricultural Sciences, Wanzhou, Chongqing 404155, China; Institute of Rice, Fujian Academy of Agricultural Sciences, Fuzhou 350018, China

**Keywords:** rice blast, *R* gene, *Pid3*, map-based cloning, CRISPR/Cas9

## Abstract

Rice blast is a destructive rice disease caused by the fungus *Magnaporthe oryzae*. Here, we identified a resistance gene from the rice cultivar Wanhui 66 which is resistant to the rice blast Guy11 isolate. Genetic mapping positioned a blast resistance locus to chromosome 6. Employing map-based cloning approach ultimately mapped the novel blast resistance locus to a genomic region of 117 kb that contains the *Pid3* gene. Candidate gene prediction and cDNA sequencing indicate that the target resistance gene in the Wanhui 66 is allelic to *Pid3*, thus it was designated *Pid3-1*. Further analysis showed that the *Pid3-1* has 3 nucleotide substitutions, resulting in 3 amino acid substitutions in the Pid3-1 protein, which significantly affect the structure of the Pid3-1 protein as indicated by the 3D structure simulation. The CRISPR/Cas9 system was employed to generate a *Pid3-1* knockout mutants that confirmed that the Wanhui 66 resistant phenotype is controlled by *Pid3-1*. A molecular marker, *Indel-6-34*, cosegregates with *Pid3-1* was identified that could have a great impact on rice breeding against blast disease resistance.

## Introduction

Rice (*Oryza sativa* L.) is a staple food source for more than half of the world's population, and its yield security is directly related to global food security (Elert 2014). However, rice blast, a devastating disease caused by the filamentous fungal pathogen *Magnaporthe oryzae*, is a serious threat to the stability of agricultural production systems (Kamarudin et al. 2023), resulting in an annual loss of about 10–30% of the global rice production (He et al. 2014; Zhou et al. 2023). With the backdrop of increasing climate change and the rapid evolution of pathogens, traditional chemical control and agronomic management methods have proven insufficient to meet the demands of sustainable agricultural development (Annegowda et al. 2021; Kou et al. 2024). Therefore, it has become the core proposition in the field of plant pathology and crop genetic improvement to explore endogenous rice resistance gene resources, develop functional markers, and create new varieties with broad spectrum and persistent resistance through molecular breeding technology (Mutiga et al. 2023; Greenwood et al. 2024).

Since the gene-to-gene hypothesis was proposed in the middle of the 20th century, scientists have gradually realized that plant disease resistance is essentially the result of specific interaction between host resistance genes (*R* genes) and pathogenic avirulent genes (*Avr* genes) (Li et al. 2019). Due to its high genetic operability and economic importance, the interaction system between rice and blast fungus has gradually developed into a model system for the study of plant–pathogen interaction (Li et al. 2019). In early studies, employing classical genetic approaches identified more than 100 blast resistance sites, and more than 50 major resistance genes were successfully cloned (Greenwood et al. 2024 ; Kou et al. 2024), revealing the central role of NBS-LRR (nucleotide binding site-leucine-rich repeat) proteins in pathogen recognition (Zhou et al. 2019; Yang et al. 2025). These genes encode intracellular immune receptors that specifically recognize effector proteins secreted by pathogens and then activate multilayered defense responses including reactive oxygen species bursts, callose deposition, and systemically acquired resistance (Zhao et al. 2018; Zhai et al. 2019; Yuan et al. 2021).

Although significant progress has been made in cloning the resistant *R* genes, there are still several key scientific issues need to be further explored. Firstly, the resistance spectrum and duration of most cloned *R* genes often do not meet the production needs, and the molecular evolution trajectory and co-evolution mechanism of pathogens remain unclear (Deng et al. 2017; Yang et al. 2019). Secondly, the promotion and application of a single major gene can lead to often resistance loss within 3–5 years due to changes in the toxic structure of pathogen populations, highlighting the production risks associated with the narrow genetic basis of disease resistance (Deng et al. 2017). Moreover, the molecular basis of the “fitness cost” phenomenon of resistance genes, where increased disease resistance is often accompanied by growth and development inhibition, has not been fully resolved, limiting the co-improvement of resistance, high-yield, and high-quality traits (Yang et al. 2019).

Studies have shown that different alleles in a locus have different species-specific spectra (Greenwood et al. 2024). For example, seven alleles with different resistance profiles were identified at *Pik* resistance sites (Yoshida et al. 2009; Kanzaki et al. 2012). Therefore, it is crucial to explore and isolate more resistance genes and their alleles. The rice blast resistance gene *Pid3* was successfully cloned in 2009 (Shang et al. 2009), however, among the more than 20 reported *Pid3* alleles, only *Pi25* and *Pid3-I1* were identified via map-based cloning (Chen et al. 2011; Xu et al. 2014; Inukai et al. 2019).

In this study, a blast resistance gene was identified from the *Indica* rice variety Wanhui 66, which was resistant to rice blast bacteria, and based on nucleotide polymorphism it was designated *Pid3-1*. In contrast to the Pid3 protein sequence, *Pid3-1* encodes an NBS-LRR protein with 3 amino acid changes. Using CRISPR/Cas9 genome editing technology, we knocked out *Pid3-1* in the Wanhui 66 and found that the knocked mutants lost resistance, indicating that *Pid3-1* conferred resistance to rice blast. Additionally, we also developed a molecular marker, *Indel-6-32*, that is closely linked to *Pid3-1*. These results indicate that *Pid3-1* may have promising application prospects in rice blast resistance breeding.

## Materials and methods

### Plant materials

The *Japonica* Lijiangxintuanhegu (LTH) variety was kept in the Rice Research Institute, Fujian Academy of Agricultural Sciences. Wanhui 66 is a disease-resistant restorer line selected by the Chongqing Three Gorges Academy of Agricultural Sciences.

### *Magnaporthe oryzae* isolation

The rice blast fungus (*M. oryzae*) Guy11 isolate was provided by the State Key Laboratory for Ecological Pest Control of Fujian and Taiwan Crops, Fujian Agriculture and Forestry University.

### Inoculation and resistance evaluation of rice blast

The rice seedlings of 3–4 leaves were moved to the high humidity inoculation chamber, and the spore suspension of the *M. oryzae* Guy11 isolate was sprayed on the plants. The inoculated plants were then transferred to a plastic box covered with a wet sponge and kept in a dark room (95% to 100% RH, 25 °C) for 24 h, and then transferred to a greenhouse at 25 °C to 28 °C for 6 d. Disease response was detected 1 week after inoculation with LTH as a susceptible control. Grades 0–3 were resistant, and grades 4–9 were susceptible.

### Polymerase chain reaction amplification and marker detection

DNA was extracted in advance as essentially described by Murray and Thompson (1980), with slight modifications. DNA polymerase chain reaction (PCR) amplification was performed as previously described by Yang et al. (2020b).

### Bulked segregant analysis

Bulked segregant analysis was used to search for markers linked to target genes. The leaf DNA of 20 susceptible lines randomly selected from the F_2_ population was used to construct the susceptible DNA pool, and the leaf DNA of 20 resistant lines randomly selected from the F_2_ population was used to construct the resistant DNA pool. SSR markers distributed across the rice genome were used for linkage detection, and DNA extracted from both LTH and Wanhui 66 were used as control. The band type of the marker linked to the susceptible allele was the same as that of LTH.

### Genetic mapping of the *R* gene

The mapping population was constructed by crossing the Wanhui 66 (*Indica*) with the LTH (*Japonica*). The band types of Wanhui 66 (*R R*) and LTH (*S S*) were denoted 1 and 3, respectively, and the heterozygote plants (*R S*) were denoted 2. Preliminary genetic mapping of the *R* gene was obtained using 95 susceptible individuals (*S S*) from the F_2_ population, and 578 susceptible plants (*S S*) were genotyped for fine mapping in the F_2_ population. MAPMAKER 3.0 (Lander et al. 1987) was used for linkage analysis of *R* gene loci and SSR markers, as reported by Rahman et al. (2007). The linkage maps and physical distances obtained in this study are basically consistent with those reported previously (Yang et al. 2020a).

### Physical mapping of the *R* gene

The genomic sequence of the “Nipponbare” variety released by the International Rice Genome Sequencing Project (IRGSP, http://rgp.dna.affrc.go.jp/IRGSP/index.html) was implemented for bioinformatic analysis and generating a physical map of the target *R* gene. For cloning the *R* gene, the target locus was identified by the target gene linkage markers, and the sequences were then compared using BLAST (http://www.ncbi.nlm.nih.gov/blast/bl2seq/b12.html).

### Bioinformatics analysis

Candidate genes prediction was performed based the existing sequence annotation database (http://rice.plantbiology.msu.edu/; http://www.tigr.org/). Sequences of candidate genes were annotated using the FGENESH+ and FGENESH_C gene prediction programs (http://linux1.softberry.com/berry.phtml) for annotation of exon–intron structures, TSSP (http://linux1.softberry.com/berry.phtml) and PLACE for annotation of promoter regions (http://www.dna.affrc.go.jp/PLACE), and PFAM (http://pfam.sanger.ac.uk) for identification of conserved protein domains. The PyMOL Molecular Graphics System (https://golgi.sandbox.google.com/about) was employed to predict the spatial structure of candidate genes encoding proteins. Multiple sequence alignments were made using CLUSTAL W (http://www.ebi.ac.uk/Tools/clustalw/index.html). Amino acid identity was calculated as the percentage of identical residues in two homologues divided by the total number of residues in the reference gene.

### Sequencing of candidate genes

Primer pairs were designed based on the predicted full-length sequences of candidate genes. In general, one primer was designed according to the 3'-UTR region of the candidate gene, and the other primer was designed according to the 5′-UTR region. According to the designed primers, candidate genes were amplified from Wanhui 66 and the amplified products were sequenced.

### Targeted mutagenesis of the *R* gene with CRISPR/Cas9

The CRISPR-plant database and website were used to design highly specific gRNA interval sequences for the *R* gene in the Wanhui 66 (Xie et al. 2014). After transformation, the regenerated plants were examined and analyzed by genome editing mutations of target genes. Individual lines were selected from transgenic CRISPR-edited cell lines, and specific mutations in PCR products were identified via sequencing (Xie et al. 2014). The CRISPR/Cas9 target sequence and detection primers used in this study are shown in Table 1.

**Table 1.** Primers used for synthesizing gRNA spacers and genotyping of CRISPR/Cas9-derived mutants.

Name	Sequence	Purpose
gRNAs-*Pid3-1*	5′-GCTCACTAATCGTCAAGCTTGGG-3′	CRISPR/CAS9
*Pid3-1*-F	5-AGATACCAAGCGAGAAGGAA-3′	Screening of lines
*Pid3-1*-R	5′-CCCGATCTCAGAAAAGAGGCG-3′	Screening of lines
*Pid3-1-KO-1*	GCTCACTAATTCGTCAAGCTTGGG (1 bp insertion)	Resistance phenotyping
*Pid3-1-KO-2*	GCTCACTA—CGTCAAGCTTGGG (2 bp deletion)	Resistance phenotyping

## Results

### Genetic analysis of the resistance gene

**Fig. 1.**
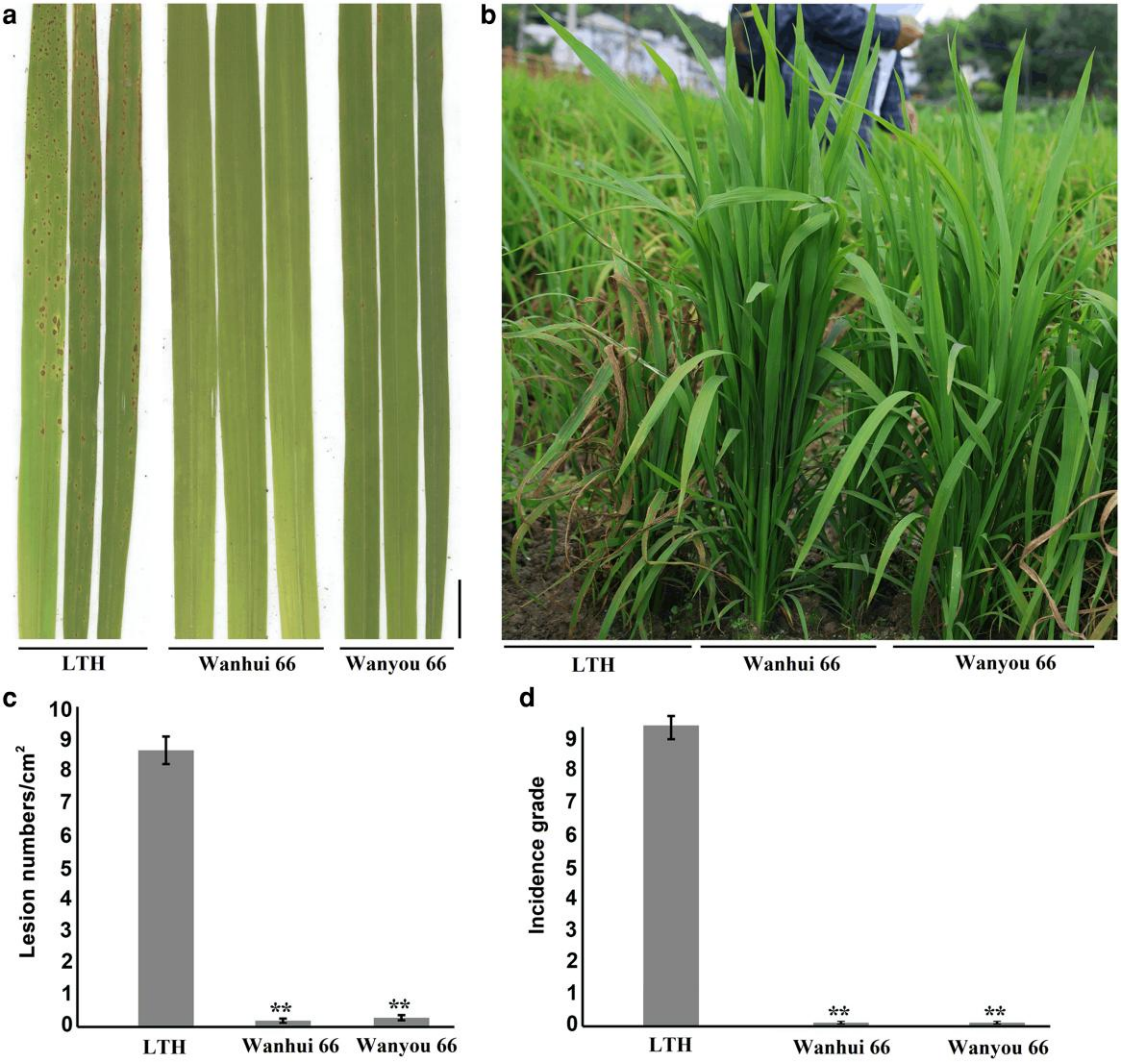
Identification of resistance of restorer line Wanhui 66 and its combination to rice blast. (a) The indoor disease assay showed that LTH was susceptible to rice blast, whereas Wanhui 66 and the combination Wanyou 66 were resistant to rice blast. Scale bar, 1 cm. (b) Field disease assay showed that LTH was susceptible to rice blast, while Wanhui 66 and the combination Wanyou 66 were resistant to rice blast. (c) Lesion numbers per cm^2^ on rice leaves (mean ± SD, *n* > 10 leaves) after indoor inoculation with blast fungus as in (a). (d) Incidence grade after field inoculation with blast fungus as in (b).

To analyze the blast resistance in the Wanhui 66 and the combination Wanyou 66 in the laboratory, 2-week-old plants growing in the greenhouse were inoculated with the blast fungus Guy11 isolate. Using susceptible rice LTH as a control, the results showed that LTH exhibited high blast susceptibility, while Wanhui 66 and Wanyou 66 showed high blast resistance (Fig. 1a and c).

In order to further analyze the field resistance levels, Wanhui 66, Wanyou 66, and LTH were grown in the National Blast Resistance Identification Center in Shanghang County, Fujian Province. The results showed that LTH demonstrated high susceptibility to rice blast, with an incidence grade of 9, whereas Wanhui 66 and Wanyou 66 were highly resistant to rice blast, with an incidence grade of 0 (Fig. 1b and d).

For the genetic analysis, the resistant parent Wanhui 66 was crossed with the susceptible parent LTH. A total of 24 F_1_ individuals exhibited a resistance phenotype against the *M. oryzae* Guy11 isolate. Indoor resistance evaluation results showed that the segregation of resistant (*R*) and susceptible (*S*) progenies in the F_2_ population fitted a 3:1 ratio (the Wanhui 66 × LTH F_2_ population, 577 *R*: 191 *S*, *χ*^2^ = 0.35; the LTH × Wanhui 66 F_2_ population, 361 *R*: 122 *S*, *χ*^2^ = 0.30, Table 2). The *R*/*S* ratio showed that Wanhui 66 contained a dominant resistance gene.

**Table 2.** Resistance of Wanhui 66 and the offspring hybrid to the rice blast Guy11 isolate.

Cross	F_1_ Phenotype	F_2_ population			*χ* ^2^ (3:1)	*P*
		Resistant individuals	Susceptible individuals	Total plants		
Wanhui 66 × LTH	Disease resistant	577	191	768	0.35^a^	>0.9
LTH × Wanhui 66	Disease resistant	361	122	483	0.30^a^	0.5–0.75

Note: ^a^Denotes that the segregation ratio of resistant plants to susceptible plants complied with 3:1 at the 0.05 significance level.

### Genetic mapping of the resistance locus

To determine the genetic position of the locus responsible for the resistance phenotype in Wanhui 66, 506 SSR markers were screened for the development of the rice genetic map. Out of these, 271 primer pairs exhibited polymorphisms between Wanhui 66 and LTH. These 271 pairs of primer were then implemented for linkage analysis in the F_2_ population (Wanhui 66 × LTH) using 20 resistant DNA and 20 susceptible DNA pools. Each of the 271 primer pairs examined 4 samples, ie, Wanhui 66, LTH, the 20 resistant DNA pool, and the 20 susceptible DNA pool.

When using *RM3330* and *RM3207* markers, the test results showed that Wanhui 66 exhibited the same DNA profile as the resistant DNA pool, whereas LTH exhibited the same DNA profile as the susceptible DNA pool. Therefore, we speculate that the resistance gene in Wanhui 66 may be associated with the *RM3330* and *RM3207* markers.

**Fig. 2.**
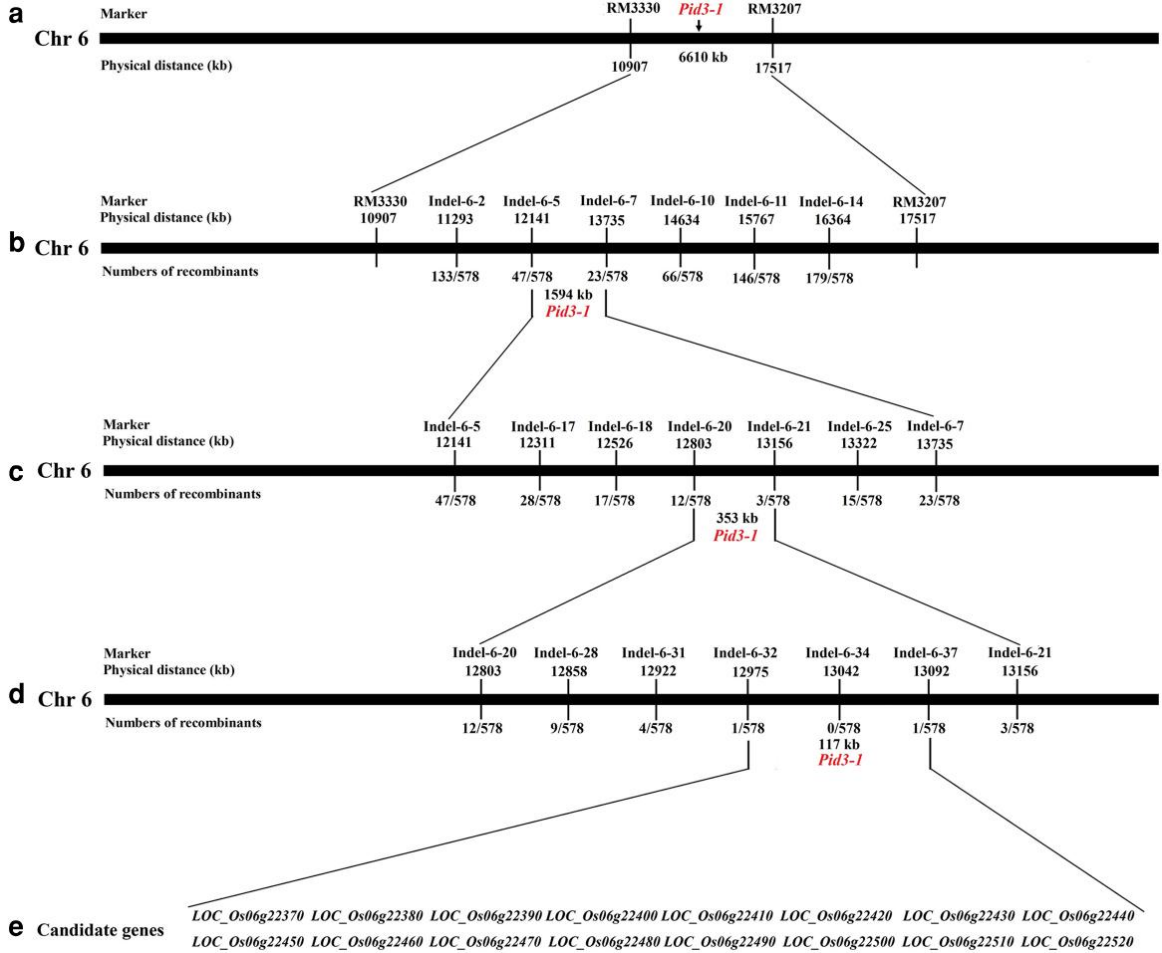
Genetic and physical maps of the *Pid3-1* gene. (a) Primary mapping of the resistance gene; the *Pid3-1* gene was identified between markers *RM3330* and *RM3207*. (b) Further genetic mapping positioned the *Pid3-1* gene between the *Indel-6-5* and *Indel-6-7* markers. (c) Fine mapping of the resistance gene; the *Pid3-1* gene was identified between the *Indel-6-20* and *Indel-6-21* markers. (d) High-resolution mapping eventually mapped the *Pid3-1* gene to a 113 kb region between the *Indel-6-32* and *Indel-6-37* markers. (e) Sixteen genes were annotated in the 117 kb region.

To preliminarily locate this resistance gene, 95 susceptible individual plants were used for further validation. The results demonstrated that *RM3330* and *RM3207* markers are linked to the resistance locus. Therefore, the resistance locus was positioned between the markers *RM3330* and *RM3207* on chromosome 6, with an estimated physical distance of about 6,610 kb (Fig. 2a).

### Genetic fine mapping of the resistance gene

To narrow down the genetic map of the resistance gene to a smaller genomic region, 578 susceptible individual plants were identified from the F_2_ population (Wanhui 66 × LTH). Further mapping was performed by using published markers (http://archive.gramene.org/markers/), and 6 Indel markers (*Indel-6-2*, *Indel-6-5*, *Indel-6-7*, *Indel-6-10*, *Indel-6-11*, and *Indel-6-14*) selected from 15 markers located between the *RM3330* and *RM3207* SSR markers. Using these 6 markers, the resistance gene was mapped at between the *Indel-6-5* and *Indel-6-7* indel markers with a physical distance of 1,594 kb (Fig. 2b and Table 3).

**Table 3.** Indel and SSR markers used for genetic fine mapping of the Pid3-1 gene.

Marker	Sequence of forward primer	Sequence of reverse primer	Physical distance (kb)	SNP locations
RM3330	CGTTCGAGCAGAACCATCTACC	CCTCTTCCGCTCCACTCTCC	10907	–
RM3207	TCCTTCATCTCCTTCGAGTCACC	ACTAAGATGGGAGGTGGGTTTCG	17517	–
Indel-6-2	ATGATTTCACTTGGCGGATGG	ACCATCACGACAGTACGATAGGG	11293	2 pb deletion
Indel-6-5	GCGGCTATGGAGGTGGATATGG	CCACCACCGTATCCTTTGTTGTATCC	12141	7 pb deletion
Indel-6-7	AGCAGATATCACACACAGCATTGG	GGAGCTTCATTTGTGATGAACCTAGC	13735	6 pb deletion
Indel-6-10	TGCACTATTGGCAGTAACATCG	GATGTGGATGGTATGAGAGTTGG	14634	6 bp replacement
Indel-6-11	ATCAATTTACTGGGTCGCACATGG	ATGATTTGACCGGGCTTTGACC	15767	4 bp replacement
Indel-6-14	CCATTTCCAGATGACTCGGATGG	AAGGCTCGTCCTCGCCTAGC	16364	3 bp replacement
Indel-6-17	GCTTCTTGTCTCTCAGGATG	TATACAAGCTAGGGATTGCC	12311	18 bp insert
Indel-6-18	CCAGTAGAAGAAAAAGGGGT	GGTTTCTCGATCCTCTCTCT	12526	39 bp deletion
Indel-6-20	CTCTCAGTTTCCACCAACTC	AAAAGGAGTATTAATTATTG	12803	21 bp insert
Indel-6-21	GCAACTAGTAGGCTTCCTCA	TCCCATACTCTGACACAAAA	13156	25 bp deletion
Indel-6-25	TCAATTTTTCCAAACCAACT	AACTCAGTTCGGTGGTTTTA	13322	34 bp deletion
Indel-6-28	ATTTTCATAACACACCTGGG	AGGTCTTGATCGATTTGATG	12858	72 bp deletion
Indel-6-31	TATATGTTCTCACGTGGGGT	TTGGGTCTGAGTAAACGAGT	12922	43 bp insert
Indel-6-32	CATCAGTTAAATTTTTAGGA	TATCAGAGCCCAAGGTAATG	12975	39 bp insert
Indel-6-34	GCATGACAGCGTTGTTCTA	TTATTTCAAATTCTGAACCT	13042	9 bp insert
Indel-6-37	GTAACCAGAATGTGGTGCTT	CTCAAAAGCTTAGCCAGAAC	13092	28 bp insert

To further delimit the physical region of the resistance gene, five Indel markers (*Indel-6-17*, *Indel-6-18*, *Indel-6-20*, *Indel-6-21*, and *Indel-6-25*) were selected from 10 new markers located between *Indel-6-5* and *Indel-6-7*. Indel marker development referred to the insertion or deletion of nucleotide fragments of different sizes at the same site of the genome between related species or different individuals of the same species. Five markers (*Indel-6-17*, *Indel-6-18*, *Indel-6-20*, *Indel-6-21*, and *Indel-6-25*) were employed for recombinant screening, revealing 28, 17, 12, 3, and 15 recombinant events, respectively. The resistance gene was found to be located between the *Indel-6-20* and *Indel-6-21* markers on chromosome 6, with a physical distance of 353 kb between the two markers (Fig. 2c and Table 3).

For further fine physical mapping, 4 polymorphic indel markers were selected from 15 new indel markers (Table 3). Five markers (*Indel-6-28*, *Indel-6-31*, *Indel-6-32*, *Indel-6-34*, and *Indel-6-37*) were employed for recombinant screening, revealing 9, 4, 1, 0, and 1 recombinant events, respectively. Therefore, the resistance gene was precisely localized within an estimated 117 kb region between the *Indel-6-32* and *Indel-6-37* markers (Fig. 2d).

### Identification of the blast resistance gene within the 117 kb region

According to the existing sequence annotation databases (http://rice.plantbiology.msu.edu/; http://www.tigr.org/), there were 16 candidate genes in the 117 kb region (Fig. 2e), and all genes had a corresponding full-length cDNA. *LOC_Os06g22370*, *LOC_Os06g22380*, *LOC_Os06g22390*, *LOC_Os06g22400*, and *LOC_Os06g22410* encode an expressed protein, whereas *LOC_Os06g22420* encodes ZOS6-04-C_2_H_2_ zinc finger protein, expressed. Gene annotations within this region revealed several putative protein-coding genes and transposable elements. The protein-coding genes include a SAM-dependent carboxyl methyltransferase (LOC_Os06g22440), a disease resistance protein RPM1 (LOC_Os06g22460.1), and a sphingolipid C4-hydroxylase SUR2 (LOC_Os06g22490.1). Additionally, multiple transposable elements were identified, encompassing CACTA/En-Spm subclass transposons (LOC_Os06g22430 and LOC_Os06g22510.1), Ty3-gypsy subclass retrotransposons (LOC_Os06g22480.1 and LOC_Os06g22520.1), and an unclassified retrotransposon (LOC_Os06g22500.1). The remaining genes (LOC_Os06g22450.1 and LOC_Os06g22470.1) were annotated as expressed or hypothetical proteins.

**Fig. 3.**
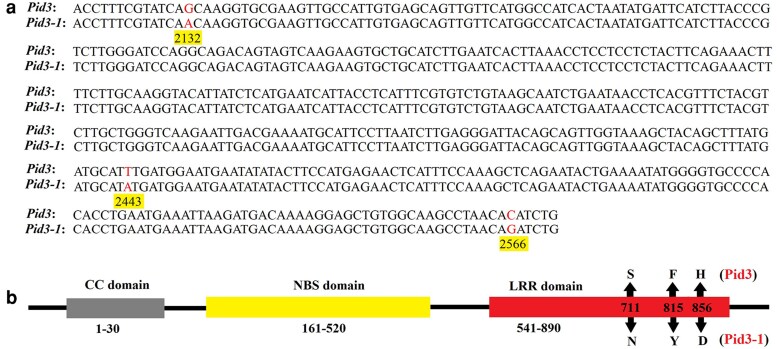
The sequence and structure comparison between *Pid3* and *Pid3-1*. (a) There were three nucleotide substitutions (G⇨A, T⇨A, and C⇨G) between *Pid3* and *Pid3-1*, with the different nucleotides highlighted and their corresponding positions indicated. (b) *Pid3* and *Pid3-1* had three identical domains (CC domain, NBS domain and LRR domain), with three amino acid substitutions (S711N, F815Y, H856D) between the Pid3 and Pid3-1 proteins.

Further analysis revealed that the *Pid3* gene, encoding NLR proteins, was located within this locus *LOC_Os06g22460* (Shang et al. 2009). To investigate which gene was responsible for the resistance phenotypes of Wanhui 66, the *Pid3* gene of Wanhui 66 was sequenced. Sequencing results identified three nucleotide substitutions (G⇨A, T⇨A, and C⇨G) in the *Pid3* gene (Fig. 3a), resulting in 3 amino acid substitution (Fig. 3b). These results indicate that the resistance gene in Wanhui 66 was most likely allelic to the *Pid3* gene. Since *Pid3* confers blast resistance and three amino acid substitution may not affect its function, we hypothesized that a *Pid3* variant may confer for blast resistance in Wanhui 66. We therefore designated this gene *Pid3-1* in Wanhui 66, and the full-length CDS sequence of *Pid3-1* is shown in Supplementary Material 1.

**Fig. 4.**
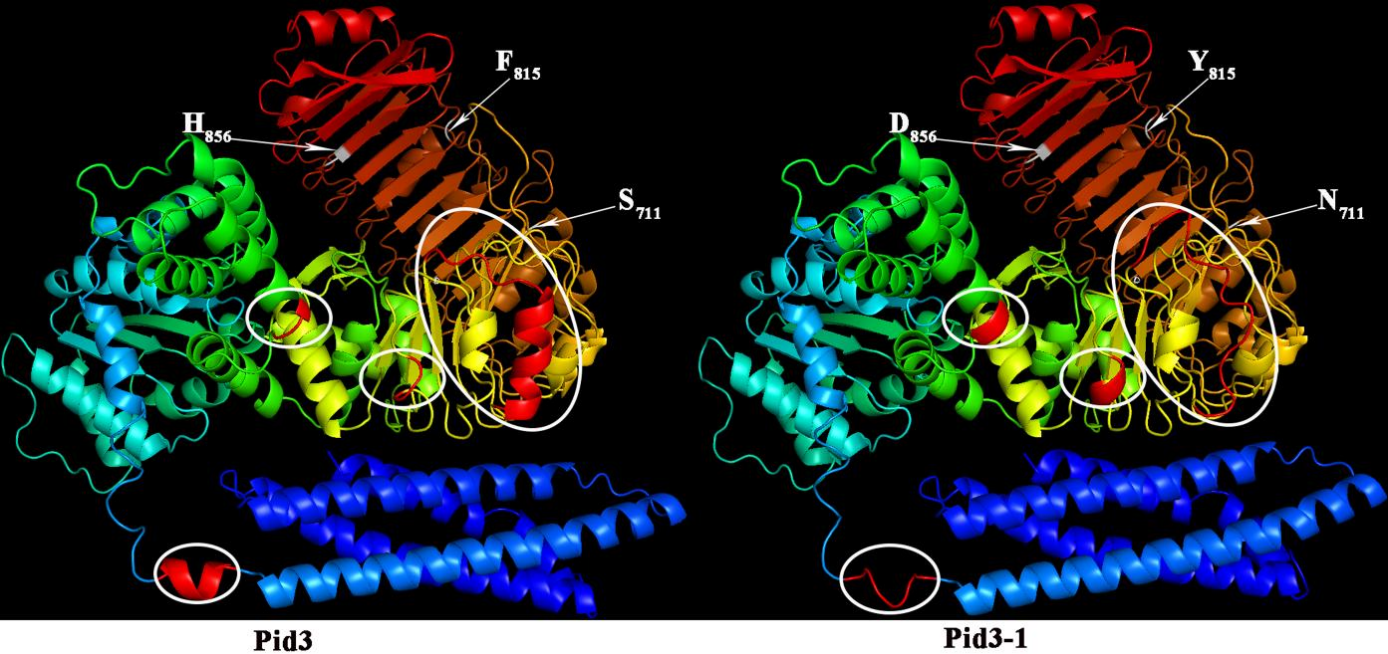
The 3D structures of the Pid3 protein and the Pid3-1 protein. Significant structural changes observed using the PyMOL Molecular Graphics System, with the 711, 815, and 856 amino acids of Pid3-1 protein are replaced by S, F, and H to N, Y, and D, respectively. These amino acid substitutions significantly affect the protein structure. The white circle indicates that the protein structural variation is caused by substitutions at the 711th, 815th, and 856th amino acids.

A preliminary analysis of *Pid3-1* protein revealed that changes in amino acids can affect protein structure. To further investigate whether replacing 3 amino acids will affect the structure of Pid3-1 protein, we simulated the spatial structure of Pid3-1 and Pid3 proteins using the PyMOL Molecular Graphics System. We observed a structural change in the LRR domain between the Pid3 and Pid3-1 proteins (Fig. 4), indicating that S_711_, F_815_, and H_856_ are critical sites of the Pid3 protein. Besides, the mutations N_711_, Y_815_, and D_856_ in Pid3-1 may partially affect the function of the Pid3-1 protein, such as its specificity.

### Functional analysis of *Pid3-1*

**Fig. 5.**
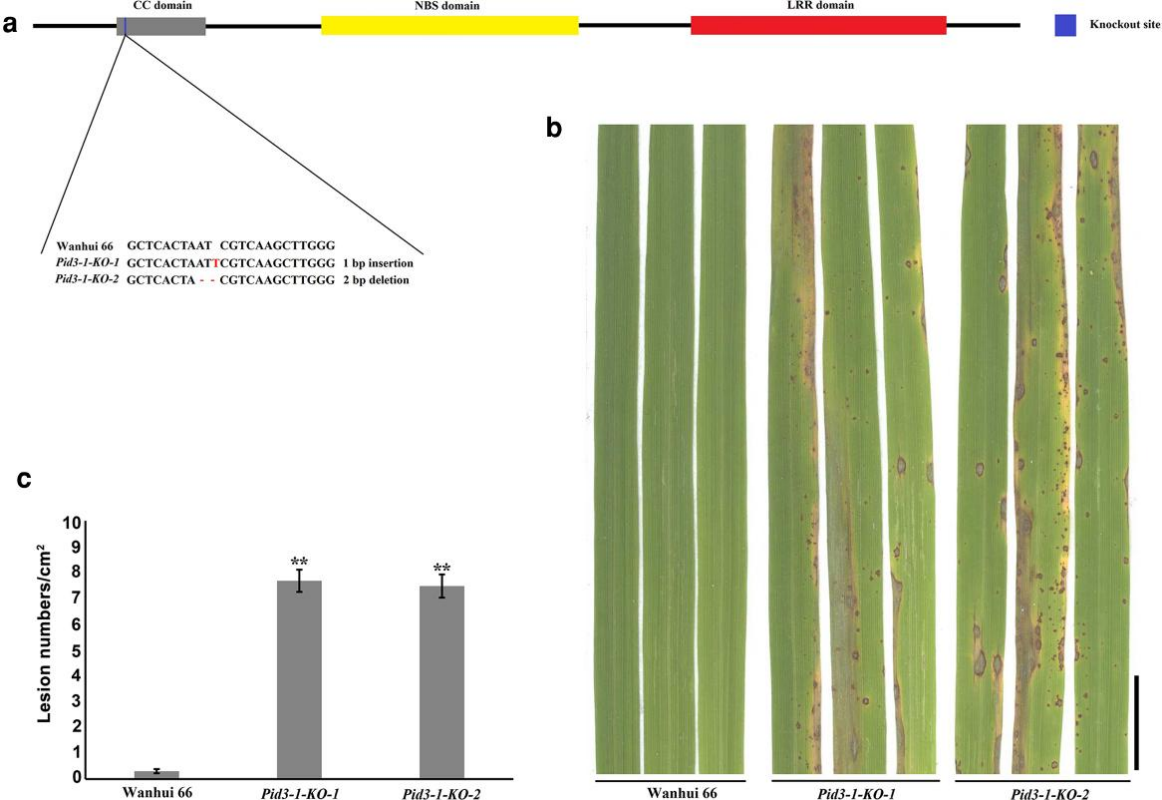
*Pid3-1* knockout lines exhibited increased susceptibility to the Guy11 isolate. (a) Two independent transgenic lines (*Pid3-1-KO-1* and *Pid3-1-KO-2*) were generated using the CRISPR/Cas9 system and verified by sequencing. (b) Inoculation with the rice blast fungus Guy11 isolate showed that the two CRISPR/Cas9-deived knockout mutants are susceptible to the Guy11 isolate, while their parent Wanhui 66 is resistant to Guy11. Scale bar, 1 cm. (c) The average numbers of lesions per cm^2^ on the rice leaves (mean ± SD, *n* > 10 leaves) after inoculation with blast fungus Guy11 isolate as indicated in (b).

To confirm the resistance function of *Pid3-1*, we investigated whether knocking out *Pid3-1* in the resistant cultivar Wanhui 66 would result in a susceptible phenotype. Therefore, the CRISPR/Cas9 gene editing system was employed to design a sequence-specific guide RNAs (gRNAs) of *Pid3-1* in the second exon. Two transgenic plants were obtained from an independent event, each with a 1 bp insertion or 2 bp deletion between at the targeted site as confirmed by sequencing (Fig. 5a and Table 1).

Subsequently, two homozygous transgenic plants from each of the two CRISPR/Cas9-derived mutants were inoculated with Guy11. The results indicate that both transgenic lines were fully susceptible to the Guy11 (Fig. 5b and c). Therefore, the targeted mutation of *Pid3-1* in the resistant Wanhui 66 resulted in susceptibility to the Guy11 isolate, demonstrating that *Pid3-1* is responsible for blast resistance in Wanhui 66.

### Identification of molecular markers closely linked to *Pid3-1*

To expedite the utilization of *Pid3-1* in enhancing rice disease resistance, we sought to identify molecular markers closely linked to *Pid3-1*. Previous studies have shown that three markers (*Indel-6-32*, *Indel-6-34*, and *Indel-6-37*) are tightly linked to *Pid3-1* (Fig. 2d). In this study, we employed these three markers to investigate polymorphisms among commonly cultivated varieties such as Nipponbare, 9311, Minhui 86, and Wanhui 66. The results revealed that the *Indel-6-34* marker was polymorphic in Nipponbare, 9311, Minhui 86, and Wanhui 66 (Supplementary Fig. 1). Conversely, the *Indel-6-32* and *Indel-6-37* markers exhibited polymorphisms only between Nipponbare and Wanhui 66. Therefore, we speculated that the *Indel-6-34* could be used as a *Pid3-1* tightly linked molecular marker, demonstrating a strong utility for rice resistance to blast disease.

## Discussion

### Genetic and evolutionary analyses of *Pid3-1*

The identification of *Pid3-1* adds a new variant to the allelic series of the *Pid3*/*Pi-25* blast resistance locus. While previous studies established that *Pi-25* and *Pid3* confer resistance to specific blast strains (Shang et al. 2009; Chen et al. 2011), our discovery of *Pid3-1* in Wanhui 66, which exhibits high resistance to the fungus Guy11, highlights the ongoing evolution and functional diversification within this locus. This finding situates *Pid3-1* within the broader genetic context of NBS-LRR gene evolution, which is characterized by mechanisms such as gene duplication and positive selection that generate novel resistance specificities (Monteiro and Nishimura 2018; Liang et al. 2025).

A central question emerging from our study concerns the functional implications of the amino acid substitutions that distinguish Pid3-1 from Pid3. Our structural simulations indicate that the residues S_711_, F_815_, and H_856_ are critical for the Pid3 structure, and their substitution to N_711_, Y_815_, and D_856_ in Pid3-1 induces significant conformational changes (Fig. 4). This observation allows us to move beyond merely describing the sequence variation to hypothesizing its functional consequences. Given that NLR proteins like Pid3 function by sensing pathogen effectors (Cesari 2018; Zhai et al. 2022), we postulate that these structural alterations in Pid3-1 may modulate its interaction interface, potentially affecting its binding affinity for specific effectors or host accessory proteins. This could underlie differences in resistance spectra or signaling intensity among these alleles.

Therefore, the sequence divergence between *Pid3-1*, *Pid3*, and *Pi-25* provides a compelling genetic foundation for investigating functional divergence. Future work should focus on constructing single-gene near-isogenic lines to systematically compare their resistance profiles against a diverse array of blast isolates. Such an approach will be crucial for deciphering whether the evolutionary path of the *Pid3* locus has led to nuanced functional specializations, thereby strengthening the rationale for deploying specific alleles in targeted resistance breeding programs.

### Analysis of the application prospects of *Pid3-1*

The enduring effectiveness of a resistance gene in agricultural production is a paramount yet often elusive breeding objective. In this context, the *Pid3-1* allele presents a compelling case for its application potential. The real-world value of this gene is strongly supported by the extensive cultivation of the restorer line Wanhui 66 and its hybrid derivative Wanyou 66 (Huang 2014), which has achieved a cumulative promotion area exceeding 66,666 hectares over the past decade. This large-scale adoption, coupled with the sustained medium resistance or better observed in the field, provides robust phenotypic evidence for the durability of the blast resistance conferred by *Pid3-1*. Our study now elucidates the genetic basis for this observed durability. The definitive confirmation of its function through CRISPR/Cas9-mediated knockout (Fig. 5) provides direct causal evidence, moving beyond correlation to firmly establish *Pid3-1* as a reliable genetic resource for durable blast resistance.

To translate this validated genetic resource into practical breeding utility, we developed the co-segregating molecular marker *Indel-6-34*. The polymorphism of this marker across diverse cultivars, including Nipponbare, 9311, and Minghui 86 (Supplementary Fig. 1), indicates its broad applicability for marker-assisted selection (MAS). This tool enables the precise introgression of the *Pid3-1* allele into elite genetic backgrounds, significantly accelerating the development of new resistant varieties.

Therefore, the combination of a genetically validated, durable resistance gene—with proven performance over a significant area—and a robust functional marker positions Pid3-1 as a highly valuable asset for future rice breeding programs. Its deployment, particularly through pyramiding with other effective *R* genes using MAS, represents a strategic approach to enhancing the spectrum and stability of blast resistance, thereby contributing to more sustainable rice production.

### Strategies for enhancing rice blast resistance breeding

The continual discovery and characterization of blast resistance genes, with over 50 major genes now cloned (Greenwood et al. 2024; Kou et al. 2024), provide a rich genetic reservoir for breeding. However, the persistent challenge of pathogen evolution necessitates more sophisticated breeding strategies to achieve durable resistance. Based on our findings and the current landscape of resistance breeding, we propose several forward-looking approaches.

Firstly, the exploration of diverse germplasm resources remains imperative. The identification of novel alleles, such as *Pid3-1* in this study, along with entirely new genes from wild relatives, landraces, and cultivated varieties via advanced genomic techniques (Kumar et al. 2022; Zhu et al. 2022 ), is crucial for expanding the genetic base of resistance.

Secondly, the functional characterization of *R* genes must include rigorous assessment of their efficacy against geographically diverse blast populations. The development of near-isogenic lines for individual *R* genes provides a powerful tool for this purpose, enabling the precise evaluation of resistance spectra and the identification of genes effective against regionally predominant strains (Jin et al. 2025).

Finally, and most critically, future efforts must prioritize gene pyramiding and the exploitation of synergistic interactions between NLR genes. Stacking multiple *R* genes in a single cultivar is a proven strategy to broaden the resistance spectrum and enhance its durability. Furthermore, emerging research reveals that specific pairs of NLR genes, such as *PigmR*/*PigmS* (Deng et al. 2017) and *RGA4*/*RGA5* (Hutin et al. 2016), can function cooperatively to confer robust and potentially more resilient resistance. While major *R* genes often confer strong, race-specific resistance that can be rapidly overcome, combining them strategically offers a promising path toward stabilizing blast resistance in the face of evolving pathogen populations (Fahad et al. 2014; Li et al. 2017).

## Conclusions

In this study, the map-based cloning approach was efficiently employed to delimit the *Pid3-1* gene from the Wanhui 66 rice cultivar to a 117 kb locus that contain also the *Pid3* gene. The CRISPR/Cas9 system was employed to generate a *Pid3-1* knockout mutants that confirmed that the Wanhui 66 resistant phenotype is controlled by *Pid3-1*. Changes in the three amino acids in Pid3-1 may partially affect the function of Pid3-1 protein, such as specificity. Finally, a molecular marker, *Indel-6-34*, closely linked to *Pid3-1* was identified which could have a great impact on ice breeding against blast disease resistance.

## Supplementary Material

jkag027_Supplementary_Data

## Data Availability

The relevant data are included within the article. The raw contributions presented in the study are also included in the article/supplementary materials. For further inquiries, please contact the corresponding authors. Supplemental material available at G3 online.
