# Supplementary material for: Genetic identification of Pid3-1 and its regulatory role in promoting blast resistance in rice
Source: G3 (Bethesda). 2026 Feb 8;16(4):jkag027. doi: 10.1093/g3journal/jkag027 (PMC13042292; doi:10.1093/g3journal/jkag027)
Supplement: jkag027_Supplementary_Data [file jkag027_supplementary_data.zip › Supplemental_Fig._1_G3-2025-406499.doc]

**Supplementary Fig. 1. Identification of molecular markers closely linked to *Pid3-1*.** The marker Indel-6-34 showed polymorphism among Nipponbare, 9311, Minghui 86 and Wanhui 66. M: Marker, 1: Wanhui 66, 2: LTH, 3: Wanhui 66/LTH, 4: Nipponbare, 5: 9311, 6: Minghui 86.
